# Selective Vitamin D Receptor Activation as Anti-Inflammatory Target in Chronic Kidney Disease

**DOI:** 10.1155/2014/670475

**Published:** 2014-01-06

**Authors:** J. Donate-Correa, V. Domínguez-Pimentel, M. L. Méndez-Pérez, M. Muros-de-Fuentes, C. Mora-Fernández, E. Martín-Núñez, V. Cazaña-Pérez, J. F. Navarro-González

**Affiliations:** ^1^Research Unit, University Hospital Nuestra Señora de Candelaria, 38010 Santa Cruz de Tenerife, Spain; ^2^GEENDIAB (Grupo Español para el Estudio de la Nefropatía Diabética) and REDINREN (RD12/0021/0019), Spain; ^3^Nephrology Service, University Hospital Nuestra Señora de Candelaria, 38010 Santa Cruz de Tenerife, Spain; ^4^Clinical Analysis Service, University Hospital Nuestra Señora de Candelaria, 38010 Santa Cruz de Tenerife, Spain

## Abstract

Paricalcitol, a selective vitamin D receptor (VDR) activator used for treatment of secondary hyperparathyroidism in chronic kidney disease (CKD), has been associated with survival advantages, suggesting that this drug, beyond its ability to suppress parathyroid hormone, may have additional beneficial actions. In this prospective, nonrandomised, open-label, proof-of-concept study, we evaluated the hypothesis that selective vitamin D receptor activation with paricalcitol is an effective target to modulate inflammation in CKD patients. Eight patients with an estimated glomerular filtration rate between 15 and 44 mL/min/1.73 m^2^ and an intact parathyroid hormone (PTH) level higher than 110 pg/mL received oral paricalcitol (1 **μ**g/48 hours) as therapy for secondary hyperparathyroidism. Nine patients matched by age, sex, and stage of CKD, but a PTH level <110 pg/mL, were enrolled as a control group. Our results show that five months of paricalcitol administration were associated with a reduction in serum concentrations of hs-CRP (13.9%, *P* < 0.01), TNF-**α** (11.9%, *P* = 0.01), and IL-6 (7%, *P* < 0.05), with a nonsignificant increase of IL-10 by 16%. In addition, mRNA expression levels of the TNF**α** and IL-6 genes in peripheral blood mononuclear cells decreased significantly by 30.8% (*P* = 0.01) and 35.4% (*P* = 0.01), respectively. In conclusion, selective VDR activation is an effective target to modulate inflammation in CKD.

## 1. Introduction 

Cardiovascular (CV) disease (CVD) is a very common feature in patients suffering from chronic kidney disease (CKD), particularly in those with end-stage renal disease (ESRD). The elevated CV morbidity and mortality in this population are not completely explained by the classical CV risk factors [1]. Nontraditional CV risk factors are essential elements in the spectrum of CVD in the renal patient, with a great relevance for the role of inflammation and the disturbances of mineral metabolism, which are already evident in the early stages of CKD [2, 3]. Both alterations contributed to the increased CV risk observed in CKD patients with secondary hyperparathyroidism, a frequent complication resulting from the loss of the ability of the kidney to regulate phosphatemia and synthesize calcitriol, the active form of vitamin D [4–6].

In the last decade, one of the most relevant therapeutic innovations in the field of CKD has been the introduction of paricalcitol (19-nor-1,25-dihydroxyvitamin D_2_), a selective activator of the vitamin D receptor for the prevention and treatment of secondary hyperparathyroidism [7, 8]. Paricalcitol has been related to a survival advantage in long-term hemodialysis patients [9, 10]. This compound acts more selectively repressing both parathyroid hormone (PTH) synthesis and secretion, with less hypercalcemic and hyperphosphatemic effects [11, 12].

On the other hand, although experimental *in vitro* and *in vivo* studies have shown that paricalcitol may have anti-inflammatory properties, clinical data are limited [13–15] and no studies have been performed to analyze the effects of selective vitamin D receptor activation as a target for modulate inflammation in patients with CKD before the initiation of dialysis treatment. Therefore, the aim of this pilot study was to evaluate the effect of the administration of oral paricalcitol to CKD patients on the serum and gene expression profile of inflammatory cytokines.

## 2. Subjects and Methods

### 2.1. Subjects and Study Design

This was a prospective, nonrandomised, open-label, proof-of-concept study of patients with CKD from a single center. The protocol was approved by the Institutional Human Research Ethics Committee, and informed consent was obtained for all patients.

Patients older than 18 years with CKD stage 3b and 4 (estimated glomerular filtration rate (eGFR) of 30–44 and 15–29 mL/min/1.73 m^2^, resp.) were considered eligible if they met the following inclusion criteria: stable clinical condition, absence of diabetes mellitus, no previous treatment with vitamin D analogs or phosphate binders, serum levels of 25-(OH)-vitamin D higher than 20 ng/mL, serum calcium and phosphorus concentrations not higher than 10 mg/dL and 4.5 mg/dL, respectively, and calcium-phosphorus product lower than 45 mg^2^/dL^2^. Exclusion criteria included severe gastrointestinal disease, current smoking habit, alcohol dependence or drug abuse, known active immunologic or tumoral disease, acute inflammatory or infectious episodes in the previous 3 months, hepatitis B, C or HIV positivity, previous transplantation, and active treatment with immunotherapy or immunosuppressive medications.

After initial evaluation, eight patients with secondary hyperparathyroidism and a serum intact PTH (iPTH) concentration higher than 110 pg/mL were treated with oral paricalcitol, which was administered as soft capsules (1 *μ*g) once a day, three times a week every other day. Nine patients matched by age, sex, and stage of CKD, with iPTH <110 mg/mL were enrolled for comparative purposes as a control group. Blood samples were drawn at baseline and after 5 months of follow-up for measurement of the study variables. No patients were lost to follow-up, and therefore, all subjects finished the study.

### 2.2. General and Specific Biochemical Parameters

Serum samples were obtained after 8–10 h overnight fasting, and frozen immediately at −80°C. Routine biochemical parameters were measured using standard methods. Serum high sensitive C-reactive protein (hs-CRP) was measured by an immunoturbidimetric fully automated assay (Roche Diagnostics GmbH, Mannheim, Germany) with functional sensitivity of 0.3 mg/L (intra- and inter-assay precision, 1.6 and 8.4, resp.). Serum levels of tumor necrosis factor-*α* (TNF-*α*), interleukin (IL)-6, and IL-10 were determined by a high-sensitive ELISA method (Quantikine Human, R&D Systems, Minneapolis, USA) in a DSXTM 4 Plate ELISA Processor (Vitro SA, Spain). Minimum detectable concentrations were 0.10 pg/mL, 0.70 pg/mL, and 0.50 pg/mL, respectively. Intra- and inter-assay coefficients of variation (CV) were <10.8% in all cases.

### 2.3. Gene Expression

For analysis of gene expression RNA, was extracted from peripheral blood mononuclear cells (PBMC). Whole blood samples (2.5 mL) from the patients included in the study were collected in PAXgene Blood RNA tubes (BD, Franklin Lakes, NJ) at the same time that serum samples. Total RNA was isolated using PAXgene Blood RNA Kit (Qiagen, Valencia, CA) and stored at −80°C. Quality of extracted RNA was tested using an Experion Automated Electrophoresis System (Bio-Rad Laboratories, Hercules, CA, USA) to ensure that 28S and 18S rRNA bands were clearly evident. RNA was quantified using a Thermo Scientific NanoDrop 2000 spectrophotometer (Thermo Scientific Nanodrop, USA). The cDNA was obtained using a High Capacity RNA-to-cDNA kit (Applied Byosistems, Foster City, CA, USA) to be used in RT-PCR and in quantitative RT-PCR.

Transcripts encoding for TNF-*α*, IL-6, IL-10, and glyceraldehyde 3-phosphate dehydrogenase (GAPDH) as constitutive gene were measured by TaqMan real-time quantitative PCR (qRT-PCR) with TaqMan Fast Universal PCR Master Mix (Applied Byosistems). TaqMan Gene Expression Assays for each transcript (Hs00174128_ml [*TNF*α**], Hs00985639_mL [*IL-6*], Hs0961622_m1 [*IL-10*], and Hs99999905_m1 [*GAPDH*]) were analysed in a 7500 Fast Real-Time PCR System (Applied Byosistems). The level of target mRNA was estimated by relative quantification using the comparative method (2^−ΔΔCt^) by normalizing to *GAPDH* expression. Quantification of each cDNA sample was tested in triplicate, and a corresponding nonreverse transcriptase reaction was included as a control for DNA contamination.

### 2.4. Statistical Analysis

Data are presented as means ± standard deviation (SD), except for inflammatory parameters, which are presented as median and range. The Shapiro-Wilk *W* test was used in testing for normality. Due to non-normal distribution, serum concentrations of inflammatory parameters and gene expression ratios were logarithmically transformed for analyses and then back-transformed to their natural units for presentation. Comparisons between basal and final values were performed by Wilcoxon Matched Pairs test. *P* < 0.05 was considered statistically significant. The fold changes in the expression of the target genes were calculated with Data Assist v3.0 Software (Applied Biosystems). Remaining computations were performed using the GraphPad Prism 5 software (GraphPad Software, San Diego, CA).

## 3. Results

### 3.1. Demographic Characteristics and Mineral Metabolism Parameters

The study was conducted in 8 male patients who received paricalcitol (mean age, 47 ± 14 years; mean eGFR, 33 ± 6 mL/min/1.73 m^2^) and in 9 male patients aged 49 ± 12 years, with a mean eGFR of 34 ± 7 mL/min/1.73 m^2^, who were included in a control group. Baseline biochemical data of participants in both groups, including mineral metabolism parameters and inflammatory variables, are depicted in Table 1. There was not any significant difference between groups except for the iPTH level, which was significantly lower in subjects included in the control group, and for this reason, patients in the control group did not receive paricalcitol.

After 5 months of follow-up, serum calcium, phosphorus, and calcium-phosphorus product (CaxP) did not show significant variations in any group. However, serum iPTH concentration experienced a significant reduction from 201 (166–228) pg/mL to 100 (71–121) pg/mL (*P* < 0.01) in patients receiving paricalcitol, which represents a median reduction of 50.4%. On the contrary, this parameter showed a trend to increase in the control group, with a median increment of 3.7% (*P* = 0.08).

### 3.2. Serum Concentration of Inflammatory Variables

Regarding the comparison of the inflammatory profile, the serum concentrations of hs-CRP, TNF-*α*, and IL-6 showed a significant decrease in patients treated with paricalcitol (Table 2). The mean percent decline of these parameters from baseline to the end of the study was 13.9% (95% confidence interval (CI), −24.4 to −4.7) (*P* < 0.01), 11.9% (95% CI, −21 to −0.5) (*P* = 0.01), and 7.0% (95% CI, −13.9 to 2.5) (*P* = 0.04), respectively. The serum level of IL-10 increased by 16%, but the difference did not reach statistical significance. The balance between pro- and anti-inflammatory cytokines was evaluated by the evolution of the ratios of TNF-*α* and IL-6 to the anti-inflammatory cytokine IL-10. These ratios showed a trend to reduction, with a percent decrease of 18.5% (95% CI, −43.3 to 6.3) (*P* = 0.1) and 14% (95% CI, −42 to 14.2) (*P* = 0.09), respectively. On the contrary, serum concentrations of inflammatory parameters and cytokine ratios in patients included in the control group did not show any significant modification compared with baseline.

### 3.3. Gene Expression of Inflammatory Cytokines

**Figure 1:**
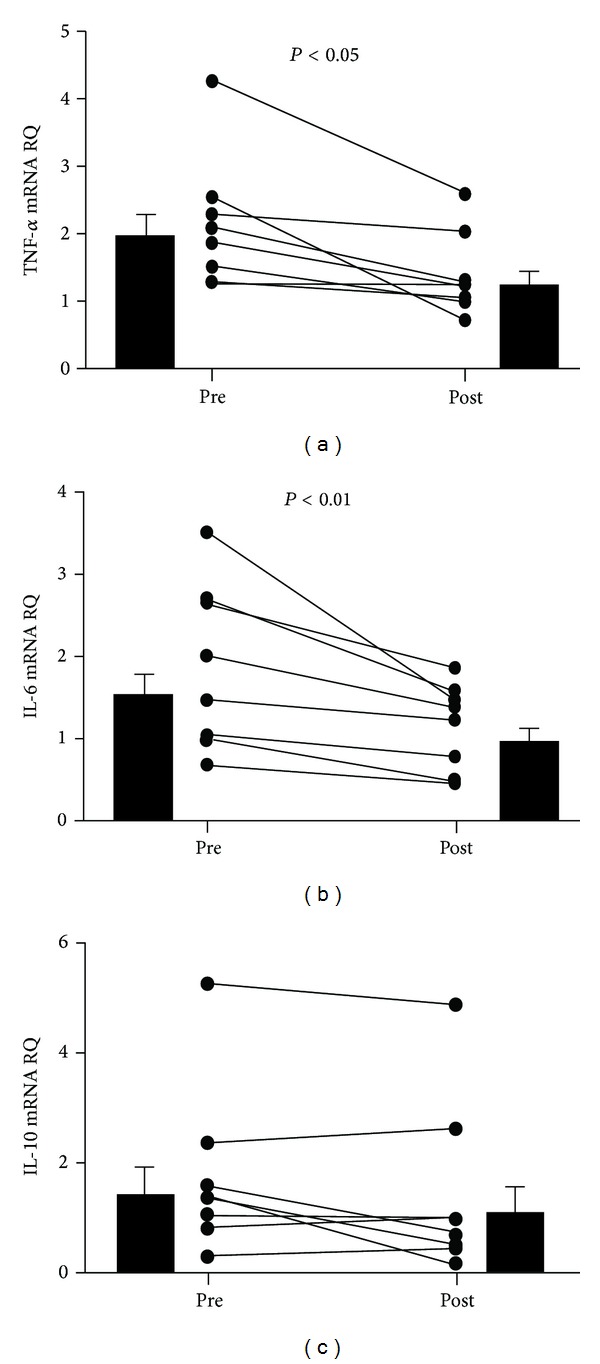
Variations in expression levels of *TNF*α**, *IL-6*, and *IL-10* mRNA in peripheral blood mononuclear cells from patients before and after treatment with paricalcitol (*n* = 8). Black circles represent means of three replicates. Black bars represent geometric mean and standard error. RQ: relative quantification.

Concerning inflammatory cytokine gene expression in peripheral blood mononuclear cells (PBMCs), the mRNA levels of TNF*α* and IL-6 decreased significantly from baseline after paricalcitol administration by 30.8% (95% CI, −49.1 to −12.6) (*P* < 0.05) and 35.4% (95% CI, −46.7 to −24.1) (*P* < 0.01), respectively, whereas expression of IL-10 did not change (Figure 1). The gene expression ratio of TNF*α*/IL-10 did not change. However, the IL-6/IL-10 ratio decreased by 35% (95% CI, −46.7 to −24.1) (*P* < 0.01). Regarding the control group, the percent variation for these parameters respect to baseline was lower than 5%, without any significant difference.

## 4. Discussion

The results of this prospective study under usual clinical practice show that selective vitamin D receptor activation with paricalcitol is an effective target to modulate the inflammatory profile of CKD patients. Specifically, paricalcitol administration was associated with a significant reduction in the serum concentrations of hs-CRP, TNF*α* and IL-6, as well as a significant decrease in the mRNA expression levels of TNF*α* and IL-6 in PBMCs.

Reduced activation of the vitamin D receptor in CKD, leading to increased expression of PTH and growth of the parathyroid glands, plays a critical role in the pathogenesis of secondary hyperparathyroidism. The usual treatment for this disorder includes the administration of active vitamin D analogs, although they have the significant disadvantage of increasing calcium and phosphorus levels. Paricalcitol (19-nor-1,25-hydroxi-vitamin D_2_), a selective, new generation vitamin D receptor activator, has been proven to be beneficial in the control of secondary hyperparathyroidism both in hemodialysis and predialysis patients with less calcemic and phosphatemic effects [16, 17]. In our study, after 5 months of paricalcitol administration, the median iPTH concentration decreased significantly by 50.4%, similar to the findings by recent studies with oral paricalcitol under usual clinical practice [17, 18].

The ubiquitous vitamin D receptor distribution in the human tissues is responsible for the pleiotropic effects of the vitamin D receptor activation beyond its classical effects [19], including significant actions on oxidative stress, immune regulation, and inflammation [20, 21]. Experimental research has shown that paricalcitol is able to modulate inflammation [22–24]. However, data regarding the potential anti-inflammatory actions of paricalcitol at the clinical level are scarce, and the studies were performed with intravenous paricalcitol or in patients on hemodialysis [13–15].

The present work is the first study specifically designed to analyze the effects of selective VDR activation as a target to modulate the inflammatory profile of CKD patients not receiving dialysis. We found that administration of paricalcitol was associated with a significant decrease in serum concentrations of hs-CRP and the proinflammatory cytokines TNF-*α* and IL-6, with a trend to reduction in the TNF-*α*/IL-10 and IL-6/IL-10 ratios. To the best of our knowledge, only one previous study has looked at potential anti-inflammatory effects of paricalcitol in CKD patients, although only serum hsCRP was assessed as an inflammatory marker (neither serum nor gene expression levels of inflammatory cytokines were evaluated). In a one-month randomized, controlled trial designed to determine whether the use of paricalcitol leaded to improvement in markers related to the progression of CKD, Alborzi et al. [25] found that hsCRP increased by 50% in the placebo group whereas it decreased in patients that received paricalcitol (20% reduction with paricalcitol 1 *μ*g/day and 30% reduction with paricalcitol 2 *μ*g/day; *P* < 0.05 between groups). The reduction of hsCRP in our study was 13.9%, which may be explained by the lower dose (1 *μ*g/48 h). Similarly to our study, the work by Alborzi et al. included 8 subjects in the study groups and most of them were males, although a significant proportion of patients were diabetics and the renal function was better (mean eGFR 47 mL/min versus 33 mL/min).

Finally, in addition to the effect on serum inflammatory profile, paricalcitol administration was associated with a significant reduction of proinflammatory cytokines gene expression in PBMCs. In a recent *in vitro* study, Eleftheriadis et al. [26] demonstrated that paricalcitol was able to reduce basal and lipopolysaccharide-induced TNF-*α* and IL-8 production by PBMCs from healthy subjects. Our study is in agreement with those previous findings and shows a decrease of mRNA expression levels of TNF-*α* and IL-6 by 30.8% and 35.4%, respectively.

Although presenting novel information, our study has some limitations. Firstly, the low number of patients may have influenced the lack of statistical differences. Secondly, this work was planned as a proof-of-concept study, and therefore the study was not performed based on a randomized design. However, based on the inclusion of a control group and the homogeneity in terms of treatment and follow-up at a single centre, without changes in the general care and therapeutic approach to the patients during the study, we think that the results can be assumed to be due to paricalcitol administration. Finally, our determinations are based on single measurement of inflammatory markers that are subject to certain variability.

## 5. Conclusions

In CKD, inflammation is a common condition where inflammatory cytokines are key molecules involved in cardiovascular injury, vascular calcification, and atherosclerosis [27, 28]. It has been demonstrated that serum CRP and IL-6 are strong and independent predictors of all-cause and CV mortality in this population [27–29]. In addition, monocytes from ESRD patients show increased expression of proinflammatory cytokines and evidence characteristics of primed prestimulated proinflammatory cells [30], a condition related to CV events [31, 32]. The results of our study demonstrate that selective vitamin D receptor activation is an effective target to modulate inflammation in CKD patients. Therefore, the immunomodulatory and anti-inflammatory effects of paricalcitol might contribute to the benefits in patient survival, hospitalizations, and mortality rates in dialysis patients [9, 10, 33], which has been suggested to be related not solely to the traditional effects of vitamin D receptor activation on bone and mineral homeostasis. Further long-term prospective trials are necessary to assess the impact of paricalcitol on clinical outcomes in CKD patients.

## Figures and Tables

**Table 1:** Demographic and baseline serum biochemistries of patients in the study and control groups.

Variable	Study group (*N* = 8)	Control group (*N* = 9)	*P* value
Age (years)	47 ± 15	48 ± 13	NS
Diabetes	3 (37.5%)	3 (33.3%)	NS
Phosphate (mg/dL)	4.2 (3.7–4.5)	4.4 (3.8–4.8)	NS
Calcium (mg/dL)	8.9 (8.3–9.7)	9.0 (8.5–9.9)	NS
Ca × P (mg^2^/dL^2^)	37 (35–42)	39 (34–43)	NS
Intact PTH (pg/mL)	201 (166–228)	136 (127–148)	<0.01
hs-CRP (mg/L)	4.9 (3.3–7.7)	4.9 (3.9–8.0)	NS
TNF-*α* (pg/mL)	7.8 (5.1–9.0)	7.6 (5.5–9.2)	NS
Interleukin-6 (pg/mL)	6.5 (2–12)	6.8 (2.8–11.2)	NS
Interleukin-10 (pg/mL)	67 (41–125)	72 (32–101)	NS

Ca × P: calcium-phosphate product; PTH: parathyroid hormone; hs-CRP: high-sensitive C-reactive protein; TNF-*α*: tumor necrosis factor-*α*; NS: not significant.

**Table 2:** Comparison of serum biochemistries in the study and control groups.

	Basal	Final	*P* value
Study group (*N* = 8)			
hs-CRP (mg/L)	4.9 (3.3–7.7)	4.5 (2.8–6.3)	<0.01
TNF-*α* (pg/mL)	7.8 (5.1–9.0)	6.3 (4.9–8.2)	0.01
Interleukin-6 (pg/mL)	6.5 (2–12)	5.9 (1.8–11.6)	<0.05
Interleukin-10 (pg/mL)	67 (41–125)	74 (52–119)	NS
Control group (*N* = 9)			
hs-CRP (mg/L)	4.9 (3.9–8.0)	4.9 (4.2–7.7)	NS
TNF-*α* (pg/mL)	7.6 (5.5–9.2)	7.4 (5.2–9.1)	NS
Interleukin-6 (pg/mL)	6.8 (2.8–11.2)	7.0 (2.4–11)	NS
Interleukin-10 (pg/mL)	72 (32–101)	66 (35–99)	NS

Ca × P: calcium-phosphate product; PTH: parathyroid hormone; hs-CRP: high-sensitive C-reactive protein; TNF-*α*: tumor necrosis factor-*α*; NS: not significant.
